# Evaluation of Hematological, Biochemical, and Coagulation Tests in Patients with Hepatitis C

**DOI:** 10.3390/medicina61112049

**Published:** 2025-11-17

**Authors:** Ieva Janulaityte, Gintare Petkute, Asta Maciuliene, Jurgita Borodiciene, Jokubas Kareiva, Astra Vitkauskiene

**Affiliations:** 1 Department of Laboratory Medicine, Faculty of Medicine, Academy of Medicine, Lithuanian University of Health Sciences, LT-44307 Kaunas, Lithuania; astra.vitkauskiene@lsmu.lt; 2 Faculty of Medicine, Academy of Medicine, Lithuanian University of Health Sciences, LT-44307 Kaunas, Lithuania; petkutegintare3@gmail.com (G.P.); jokubas.kareiva@stud.lsmu.lt (J.K.); 3 Anesthesiology Clinic, Faculty of Medicine, Academy of Medicine, Lithuanian University of Health Sciences, LT-44307 Kaunas, Lithuania; asta.maciuliene@lsmu.lt (A.M.); jurgita.borodiciene@lsmu.lt (J.B.)

**Keywords:** hepatitis C virus, non-invasive indices, hematological parameters, gender differences, viremia, fibrosis markers

## Abstract

*Background and Objectives*: Hepatitis C virus (HCV) remains a significant cause of chronic liver disease worldwide. While direct-acting antivirals achieve high cure rates, the interplay between viral load, gender, and routine laboratory parameters remains unclear. This study aimed to investigate hematological, biochemical, and coagulation profiles, as well as derived non-invasive indices, in HCV-infected patients, stratified by gender and viremia levels. *Materials and Methods*: This retrospective study included 367 patients with HCV infection (223 males and 144 females). Patients were divided into four groups: high viremia males (hiVM), high viremia females (hiVF), low viremia males (loVM), and low viremia females (loVF), using 800,000 IU/mL as the threshold. Routine hematological, biochemical, and coagulation tests were conducted, and derived indices (FIB-4, APRI, AST/ALT ratio, PLR, NLR, SII, AISI, PNI, HALP, PAR, NAR) were calculated. *Results*: Significant gender- and viremia-specific differences were observed. hiVM showed higher erythrocyte indices and altered coagulation parameters, whereas hiVF had increased lymphocyte counts and AST/ALT ratio elevation. loVM displayed reduced hemoglobin and hematocrit, along with worse coagulation results. Biochemical analysis revealed gender differences in GGT, bilirubin, and albumin levels. Among derived indices, FIB-4 and APRI were higher in loVM, while SII and PLR were elevated in loVF. At the second visit after 17±4 weeks, when patients had no detectable HCV DNA in the peripheral blood, most indices improved significantly across groups. *Conclusions*: HCV infection affects laboratory profiles depending on gender and viremia levels. Non-invasive indices from routine tests offer valuable insights into inflammatory and nutritional status. Using these indices alongside traditional markers may aid hypothesis generation or clinical assessment and help prioritize further assessment for HCV patients.

## 1. Introduction

Hepatitis C virus (HCV) remains a major global health issue, affecting over 58 million people worldwide with chronic infection. The disease often stays asymptomatic for many years, but it slowly damages the liver, leading to cirrhosis, hepatocellular carcinoma (HCC), and the need for transplantation [1]. Even though effective direct-acting antivirals can achieve a sustained virological response in over 95% of cases, only a small number of patients are diagnosed and treated. This limits progress toward the World Health Organization’s (WHO) goal of eliminating HCV by 2030 [2].

Laboratory evaluation is essential in managing HCV infection [3,4]. Routine hematological, biochemical, and coagulation tests offer vital information about systemic and liver functions, indirectly reflecting immune activity, fibrotic progression, and the extent of liver cell injury. Changes in lymphocyte counts, erythrocyte-related indices, and platelet-related markers may indicate immune dysregulation and early fibrotic changes [5]. Elevated liver enzymes, bilirubin levels, and decreased albumin levels signal liver cell damage and reduced synthetic function, while coagulation parameters reflect the liver’s role in blood clotting. Additionally, non-invasive indices based on these routine tests may help estimate the severity of fibrosis and disease progression without the need for invasive or costly procedures [5,6].

Two factors remain inadequately addressed when interpreting these laboratory markers: the level of viremia and patient gender. Viral load is clinically significant because high viremia indicates active replication, predicts more difficult treatment, and may cause greater systemic effects. Gender, in turn, influences disease progression through hormonal, immune, and metabolic pathways [7,8]. Women are often studied as a separate group in biomedical research due to complex hormonal fluctuations that can affect immune system activity and alter laboratory results. These hormonal changes—especially in estrogen and progesterone levels—are known to influence both innate and adaptive immune responses, which may explain sex-specific differences in vulnerability and host response to infections, including HCV [9]. However, women are consistently underrepresented in biomedical research, leading to a lack of gender-specific data. Clinically, this gap is compounded by women’s tendency to underreport non-specific symptoms and the difficulty of distinguishing hormonal fluctuations from disease-related changes, which can result in signs being overlooked or underestimated [10].

To date, most studies on HCV have emphasized virological and biochemical endpoints, while fewer have systematically addressed how gender and viral load modulate routine laboratory tests and derived indices. Gender differences are significant, as hormonal, metabolic, and immunological mechanisms may shape disease progression, treatment response, and long-term outcomes. Moreover, viral load not only reflects active replication but may also influence systemic hematological and inflammatory responses. Previous research has demonstrated the prognostic value of indices such as the fibrosis-4 index (FIB-4) and AST to platelet ratio index (APRI) in assessing fibrosis [11,12,13], while newer indices like Systemic Immune-Inflammation Index (SII), Aggregate index of systemic inflammation index (AISI), and hemoglobin-albumin-lymphocytes and platelets index (HALP) integrate immune and nutritional status [14,15]. However, their combined use in relation to gender- and viremia-specific patterns in HCV remains poorly explored. Addressing this gap may enhance non-invasive disease monitoring and align clinical assessment with precision medicine approaches.

The current study investigates how viremia levels and gender influence routine hematological, biochemical, and coagulation parameters, along with derived indices, in patients with chronic HCV infection. The strength of this approach lies in combining standard, affordable laboratory tests with a gender- and viremia-specific focus. This work aims to provide clinically relevant evidence for a more precise and fair assessment of HCV patients, potentially improving diagnosis, monitoring, and treatment decisions in real-world clinical settings. We hypothesize that viremia levels and patient gender significantly impact routine laboratory parameters and derived indices in HCV infection, and including these factors will improve the accuracy of patient evaluation beyond standard methods.

## 2. Materials and Methods

### 2.1. Course of the Study and Selection of Subjects

**Figure 1:**
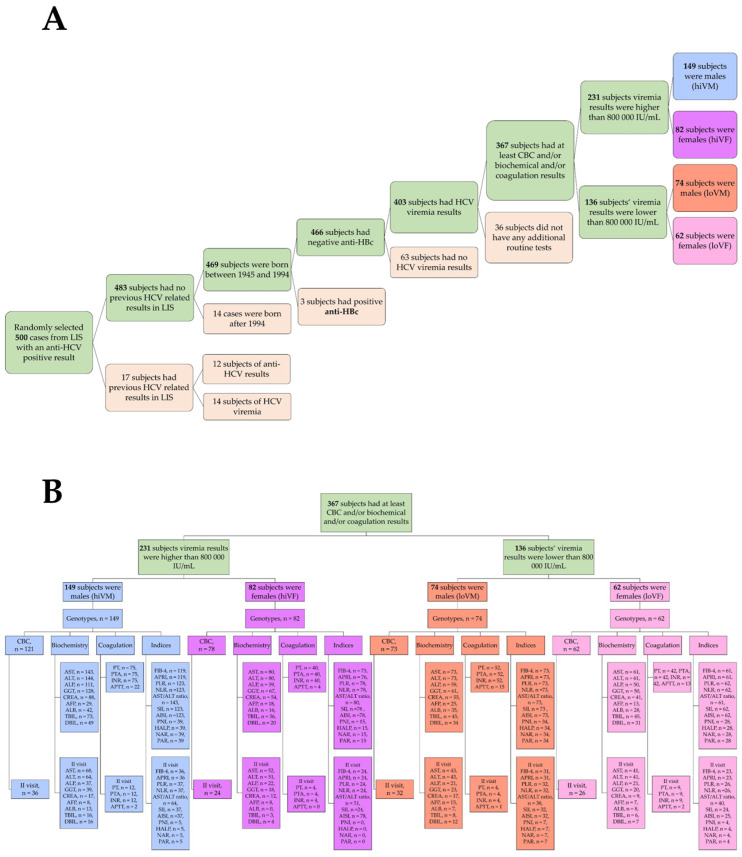
(**A**)—inclusion and exclusion rationale; (**B**)—each study group’s routine laboratory test results that are evaluated and presented in the study.

A retrospective analysis of laboratory test results from patients tested for HCV infection at the Laboratory Medicine Clinic of the Hospital of LUHS Kauno Klinikos was conducted. The study included men and women born between 1945 and 1994 who were screened under the HCV screening program between 2022 and 2024. A total of 367 patients were selected, consisting of 223 males and 144 females, who were further divided into four groups based on gender and viremia level: high viremia males (hiVM), high viremia females (hiVF), low viremia males (loVM), and low viremia females (loVF). The low and high viremia were determined by the international units per liter (IU/L). High viremia patients were those with results higher than 800,000 IU/L, and low viremia patients had results lower than 800,000 IU/L. The rationale for choosing 800,000 IU/L as a threshold was based on 2018 recommendations by the European Association for the Study of the Liver (EASL) [16]. Additional inclusion and exclusion criteria are stated in Figure 1.

Some of the subjects returned for a second visit, which was 3–6 months after the initial visit (first visit). For those subjects, the HCV viremia test was repeated and found to be 0 IU/L. Together with other hematological, biochemical, and/or coagulation tests, the reduction in viremia was assessed as a treatment effect. It is essential to note that not all patients have been subjected to all the listed indicators, as doctors have not been provided with precise and specific guidelines on which tests should be performed on patients with hepatitis C; instead, these decisions are usually based on individual symptoms. The number of tests performed on the subjects is stated in Figure 1.

### 2.2. Detection of Hepatitis C Virus Antibodies

Venous blood was collected in standard vacuum tubes without an anticoagulant—BD Vacutainer^®^ SST™ (without a separating gel) (BD, Heidelberg, Germany) or Serum Separation Tubes; BD Vacutainer^®^ Serum Tubes (with separating gel) (BD, Heidelberg, Germany). The subjects’ samples were transported to the laboratory and centrifuged according to established requirements. After centrifugation, the samples were analyzed using the ETI-MAX 3000 automatic immunological analyzer (DiaSorin S.p.A., Saluggia, Italy) with a kit of Murex anti-HCV V4.0 reagents (DiaSorin S.p.A., Italy).

### 2.3. Determination of Viremia by the In Vitro Nucleic Acid Amplification Assay

Venous blood was collected in standard vacuum tubes with EDTA—BD Vacutainer^®^ EDTA Tubes (BD, Heidelberg, Germany). The test tubes were transported to the laboratory within 24 h of sample collection and then centrifuged at 1000× *g* for 10–15 min. The plasma was then separated according to standard laboratory procedures. For nucleic acid amplification, the instruments of QIAsymphony^®^ SP/AS (QIAGEN GmbH, Hilden, Germany) and the Rotor-Gene^®^ Q MDx 5plex HRM (QIAGEN GmbH, Hilden, Germany) thermocycler were used, as well as artus HCV QS-RGQ Kit (QIAGEN GmbH, Hilden, Germany) reagents. The lower limit of detection (LLOD) of the HCV RNA quantification assay used in our study was 15 IU/mL, and the lower limit of quantification (LLOQ) was 25 IU/mL, as specified by the manufacturer. All testing was conducted according to the manufacturer’s protocol and internal laboratory quality assurance standards. After the RNA was purified, the remaining unused samples were frozen and stored at −80 °C.

### 2.4. HCV Genotyping

The frozen samples were thawed at room temperature (15–25 °C) immediately before amplification. The study used the VERSANT^®^ HCV Amplification 2.0 Kit (LiPA) (Siemens Healthineers, Forchheim, Germany) and a GeneAmp^®^ PCR System 9700 (Applied Biosystems, Foster City, CA, USA) thermocycler. For amplification, 20 μL of purified RNA was used. A PCR product of viral RNA isolated from blood plasma was used in this study. Hybridization and genotyping were performed according to the manufacturer’s protocol.

### 2.5. Hematological Examination

The blood of the subjects was collected by peripheral vein puncture into vacuum tubes containing the anticoagulants K2EDTA or K3EDTA—BD Vacutainer^®^ *EDTA* Tubes (BD, Heidelberg, Germany). Transport of the samples to the laboratory was conducted in accordance with established requirements. The samples were analyzed using an automated hematology analyzer, the BC UNICel DxH800 (Beckman Coulter, Brea, CA, USA). Based on the result of the hematological examination, various indices indicating inflammation and fibrosis were calculated. For hematological analytes, we measured the red blood cells (RBC), hemoglobin (HGB), hematocrit (HCT), white blood cells (WBC), monocytes (absolute count and percentage), lymphocytes (absolute count and percentage), neutrophils (absolute count), platelets (PLT), mean platelet volume (MPV), and plateletcrit (PCT). The normal values and full statistical description of hematological examination is presented in Table A1 and Table A2.

### 2.6. Biochemical Indicators

The blood of the subjects was taken by peripheral vein puncture in vacuum tubes without an anticoagulant—BD Vacutainer^®^ Serum Tubes (BD, Heidelberg, Germany), without an anticoagulant with a distinctive gel—BD Vacutainer^®^ SST™ Serum Separation Tubes (BD, Heidelberg, Germany) and with a coagulation activator heparin—BD Vacutainer^®^ Heparin Tubes (BD, Heidelberg, Germany). The subjects’ samples were transported to the laboratory and centrifuged according to established requirements. After centrifugation, the samples were analyzed with the AU680 automatic biochemical analyzer (Beckman Coulter, Brea, CA, USA) and the TOSOH AIA-900 automatic immunological analyzer (Tosoh Corporation, Yokkaichi, Japan). The analytes used for the analysis of this research work include alanine transaminase (ALT), aspartate aminotransferase (AST), creatinine (CREA), alkaline phosphatase (ALP), γ-glutamyl transferase (GGT), total bilirubin, direct bilirubin, albumin (ALB), and, by the immunological analyzer, α-fetoprotein (AFP). The normal values and full statistical description of biochemical examination is presented in Table A1 and Table A2 in the Appendix A.

### 2.7. Coagulation Indicators

The blood of the subjects was collected by puncturing the peripheral vein into vacuum tubes containing sodium citrate BD Vacutainer^®^ Citrate Tubes (BD, Heidelberg, Germany). The subjects’ samples were transported to the laboratory and centrifuged according to established requirements. After centrifugation, the samples were analyzed with the automatic coagulation system analyzer Stago STA R Max (Diagnostica Stago, Taverny, France). The analytes used in this research paper for analysis are prothrombin time (PT), prothrombin time activity (PTA), International Normalized Ratio (INR), and Activated Partial Thromboplastin Time (APTT). The normal values and full statistical description of coagulation examination is presented in Table A1 and Table A2 in the Appendix A.

### 2.8. Non-Invasive Hematological and Inflammation-Based Indices

In addition to conventional laboratory parameters, a panel of non-invasive hematological and inflammation-based indices derived from routine blood counts and biochemistry was calculated. Cut-off values from the published literature were applied where available. These derived laboratory indices were calculated to provide indirect insights into liver function, systemic inflammation, and nutritional–inflammatory balance. The FIB-4 and APRI scores were used as non-invasive surrogate markers of hepatic fibrosis; PLR, NLR, and SII reflected systemic inflammatory activity; and PNI, HALP, PAR, and NAR captured nutritional and immune–metabolic components. These indices are analytical proxies rather than diagnostic tools and may be influenced by factors unrelated to liver pathology. Therefore, their interpretation in this study is intended to be exploratory and hypothesis-generating, providing a laboratory-based perspective rather than a definitive clinical assessment. The formulas for calculations, as well as the potential applications of these indices in HCV patients, are presented in Table 1 and full statistical description of non-ivasive hematological and inflammation-based indices is presented in Appendix A2.

### 2.9. Statistical Data Analysis

The data obtained during the study were analyzed using the statistical analysis program GraphPad Prism 10 for Windows (GraphPad Software, Inc., San Diego, CA, USA). A test for normality was performed using the Shapiro–Wilk test. Given the small sample size, non-parametric statistical methods were used. To quantify the results of the individual group studies, the mean, standard deviation (SD), or standard error of the mean (SEM) was calculated for each group. The Mann–Whitney U and Kruskal–Wallis tests were used if the data were not normally distributed. The Wilcoxon signed-rank test was used with related samples. Statistically significant differences were considered to be results with a level of statistical reliability of *p* < 0.05.

## 3. Results

### 3.1. Characteristics of the Study Subjects

This study included 367 patients who underwent and received a positive HCV RNA test result at the Laboratory Medicine Clinic of Hospital of LUHS in 2022–2023. The investigated laboratory results belonged to a population aged 28 to 79 years, with a mean age of 55.2 ± 11.2 years, comprising 223 males and 144 females. The subjects were divided into four groups based on the level of viremia and gender: low viremia in men, low viremia in women, high viremia in men, and high viremia in women. Low viremia is defined by an HCV RNA score below 800,000 IU/mL, and high viremia is defined by more than 800,000 IU/mL. Eight genotypes/subtypes were identified in the subject population: 1, 1a, 1a/1b, 1b, 2, 2a/2c, 3, and 3a. The study group with high viremia consisted of 231 patients, comprising 149 males and 82 females. The mean age of hiVM was 53.48 ± 10.29 years, and the mean age of hiVF was 58.32 ± 10.63 years, and they were significantly older, *p* < 0.001. The age group of subjects with low viremia consisted of 74 men and 62 women. The mean age of the loVM group was 55.01 ± 10.57 years, while the mean age of the loVF group was 55.40 ± 13.78 years. Data are presented in Table 2.

In the subject population, eight genotypes were identified, and their distribution was calculated according to the identified study groups. In all study groups, genotype 1b (42.23%) was the most common, followed by genotype 3a (28.88%). Other significant genotypes, including 1a (14.99%), 2a/2c (8.45%), and 2 (3.27%), were identified. Genotypes 1a/1b and 3 have been identified less frequently, with a total frequency of less than 2%. Data are presented in Table 3.

### 3.2. Evaluation of Hematological Testing

By dividing the population based on viremia level and gender, a statistical analysis of the overall hematological test parameters was conducted to evaluate the dependence of these parameters on viremia intensity and gender. In analyzing erythrocyte count, hemoglobin, and hematocrit, significantly higher results were found in the hiVM group compared to the loVM group, *p* < 0.05. When evaluating the possible differences between the women’s groups, significantly lower hemoglobin levels were found in the loVF group compared to the hiVF group, *p* < 0.05. Data are presented in Figure 1.

Hematological test results were assessed according to the reference ranges applicable to the study, and the following changes were observed only in RBC, HGB, and HCT analytes: 25% of loVM and 21% of hiVM had lower values of RBC, while 6% loVF had higher values of RBC, *p* < 0.001. HGB and HCT were found to be lower in hiVM, loVM, and loVF but less in hiVF, *p* < 0.05. Significant differences were not found in WBC, lymphocytes, monocytes, PLT, MPV, and PCT.

There were no differences between the groups in the absolute numbers of leukocytes, lymphocytes, and monocytes; however, the percentage of lymphocytes was higher in the hiVF group compared to the loVF group, *p* = 0.0100. In the loVM, the relative number of monocytes was higher than in the hiVM, *p* < 0.0001.

**Figure 2:**
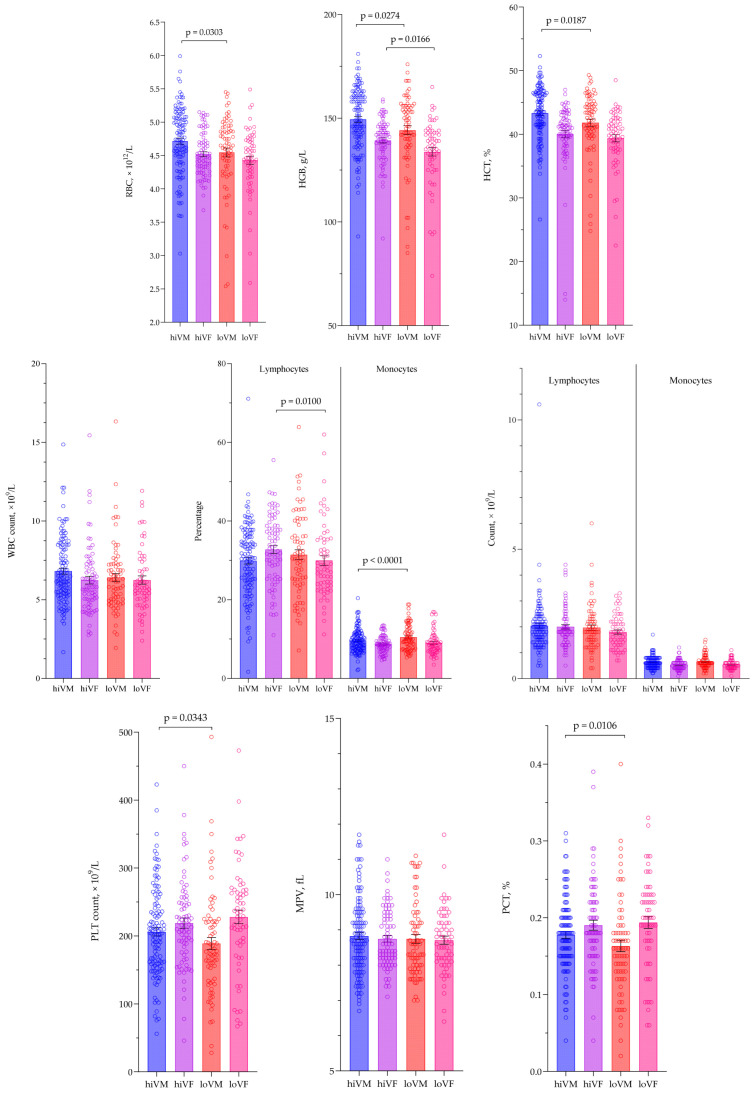
Comparison of hematological test results values between subject groups. RBC—red blood cells; HGB—hemoglobin; HCT—hematocrit; WBC—white blood cells; PLT—platelets; MPV—mean platelet volume; PCT—plateletcrit; hiVM—high viremia males; hiVF—high viremia females; loVM—low viremia males; loVF—low viremia females. hiVM n = 121, hiVF n = 78, loVM n = 73, loVF n = 62. Lines connecting comparison groups with a *p*-value denoting the significant difference and pair-wise comparisons. Statistical analysis between investigated groups—two-sided Mann–Whitney U test (independent data).

PLT count and PCT were found to be significantly lower in the loVM group compared to the hiVM group, *p* < 0.05. The MPV analysis did not show significant differences. Data are presented in Figure 2.

**Figure 3:**
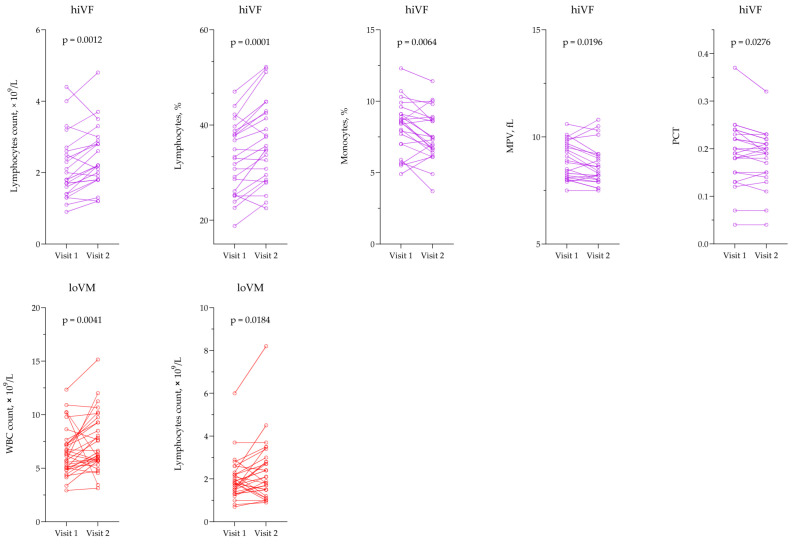
Comparison of hematological test results in the hiVF and loVM groups across different visits. hiVF—high viremia females; loVM—low viremia males; MPV—mean platelet volume; PCT—plateletcrit; WBC—white blood cells. hiVF n = 24; loVM n = 32. Lines connecting comparison groups with a *p*-value denoting the significant difference and pair-wise comparisons. Statistical analysis: Wilcoxon signed-rank test used for evaluation of differences between first and second visits.

A statistically significant increase in the number and percentage of lymphocytes and a decrease in the percentage of monocytes were observed in the hiVF group, *p* < 0.05. Furthermore, in the same group, the MPV and PCT significantly decreased, *p* < 0.05. In the loVM group, WBC and lymphocyte counts increased significantly by the second visit, *p* < 0.05. Data are presented in Figure 3.

### 3.3. Evaluation of Biochemical Parameters

**Figure 4:**
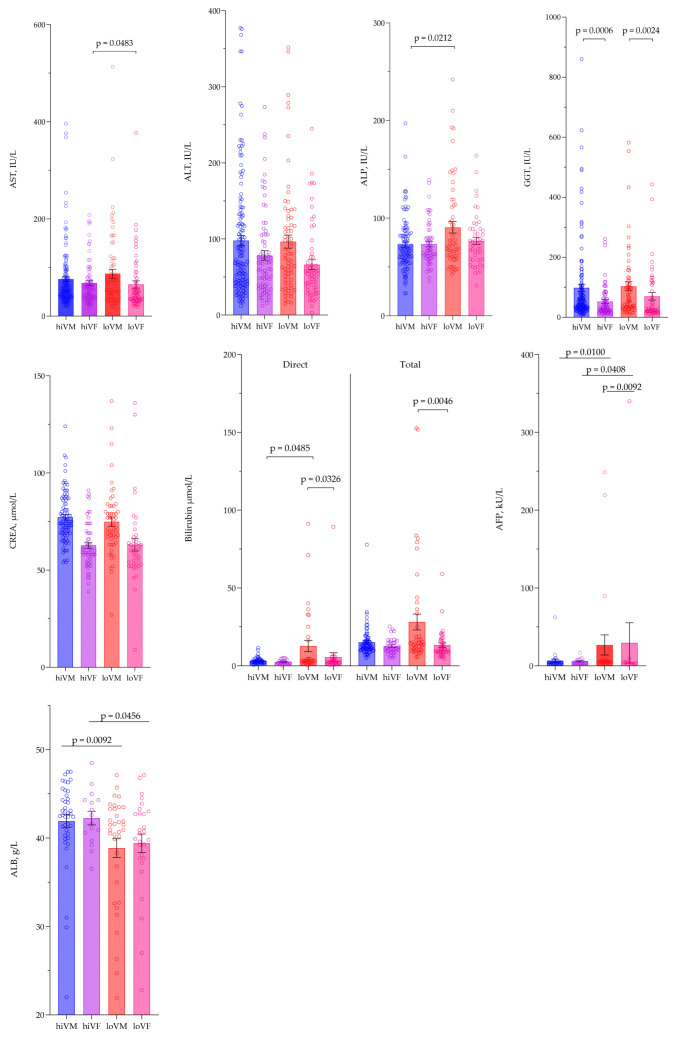
Comparison of biochemical tests between study groups. AST—aspartate aminotransferase, ALB—albumin, ALT—alanine aminotransferase, ALP—alkaline phosphatase; CREA—creatinine, GGT—γ-glutamyl transferase. AST hiVM n = 143, hiVF n = 80, loVM n = 73, loVF n = 61; ALT hiVM n = 144, hiVF n = 80, loVM n = 73, loVF n = 61; ALP hiVM n = 111, hiVF n = 59, loVM n = 59, loVF n = 50; GGT hiVM n = 128, hiVF n = 67, loVM n = 61, loVF n = 50; CREA hiVM n = 88, hiVF n = 54, loVM n = 55, loVF n = 41; direct bilirubin hiVM n = 49, hiVF n = 20, loVM n = 34, loVF n = 31; total bilirubin hiVM n = 73, hiVF n = 36, loVM n = 45, loVF n = 45; AFP hiVM n = 29, hiVF n = 18, loVM n = 25, loVF n = 13; ALB hiVM n = 42, hiVF n = 16, loVM n = 35, loVF n = 28. Lines connecting comparison groups with a *p*-value denoting the significant difference and pair-wise comparisons. Statistical analysis between investigated groups—two-sided Mann–Whitney U test (independent data).

Biochemical analytes were evaluated, including alanine aminotransferase (ALT), aspartate aminotransferase (AST), alkaline phosphatase (ALP), γ-glutamyltransferase (GGT), total and direct bilirubin, albumin, creatinine (CREA), and α-fetoprotein (AFP), among four test groups. AST was significantly higher in the hiVF group compared to the loVF group, *p* = 0.0483. ALP was significantly higher in the loVM group compared to the hiVM group, *p* = 0.0212. Meanwhile, differences in GGT were found between the genders in the same viremia groups; in the hiVM and loVM groups, the GGT was significantly higher than in the hiVF and loVF groups, *p* < 0.05. No significant differences were found between groups in ALT and CREA test results. Direct bilirubin levels were significantly higher in loVM compared to hiVM and loVF; accordingly, *p* = 0.0485; *p* = 0.0326. For total bilirubin, the loVM had significantly higher values compared to the loVF group, *p* = 0.0046. AFP levels in the loVM group were significantly higher compared to the hiVM group, *p* = 0.0100, and higher in the loVF group than in the hiVF and loVM groups, *p* < 0.05. Albumin levels were significantly lower in the loVM and loVF groups than in the hiVM and hiVF groups, *p* < 0.05. Data are presented in Figure 4.

The majority of patients had elevated values over the normal range of AST, ALT, and GGT in all study groups. For AST, the highest cases were found in females—81% of the hiVF group, 70% of the loVF group. In the male groups, the rates were 64% of the loVM and 59% of the hiVM, *p* = 0.006. For ALT, the tendencies were similar, and most cases with elevated ALT values were found in females—77% of high-value females (hiVF), 73% of low-value females (loVF), while in men, 71% of low-value males (loVM) and 66% of high-value males (hiVM). The highest values of GGT were recorded in the male groups: 64% of loVM and 63% of hiVM. In contrast, in the female groups, the values were 42% in hiVF and 40% in loVF, with a *p*-value of 0.002. In the ALP assessment, the loVM group stood out significantly, with 19% of the subjects having an elevated ALP value, whereas this was also observed in 8% of the loVF and 5% of the hiVF groups, *p* = 0.023. For other biochemical tests, no significant differences were observed between the study groups.

**Figure 5:**
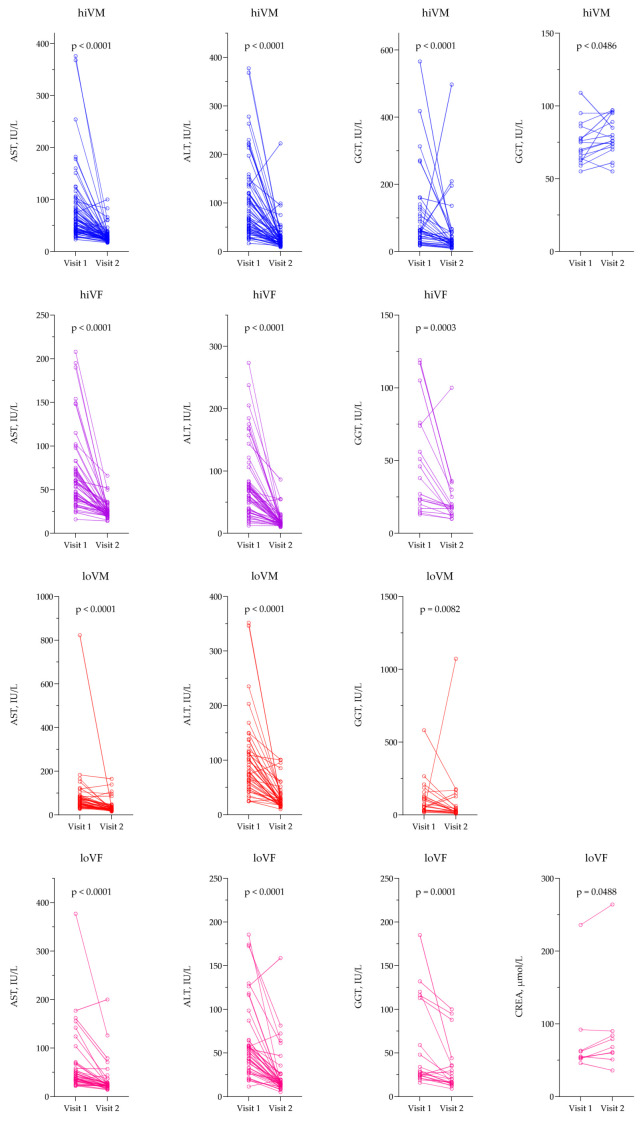
AST, ALT, GGT, and CREA comparison on different visits. ALT—alanine transaminase; AST—aspartate aminotransferase; CREA—creatinine; GGT—γ-glutamyl transferase. hiVM AST n = 68; ALT n = 64; GGT n = 39; CREA n = 17; hiVF AST n = 52; ALT n = 51; GGT n = 18; loVM AST n = 43; ALT n = 43; GGT n = 23; loVF AST n = 41; ALT n = 41; GGT n = 20; CREA n = 9. Lines connecting comparison groups with a *p*-value denoting the significant difference and pair-wise comparisons. Statistical analysis: Wilcoxon signed-rank test used for evaluation of differences between first and second visits.

For AST, ALT, and GGT, the values were lower on the second visit compared to the first visit in all study groups, *p* < 0.05. CREA was significantly higher on the second visit compared to the first visit in the hiVM and loVF groups, *p* < 0.05. Data are presented in Figure 5.

### 3.4. Evaluation of Coagulation Tests

**Figure 6:**
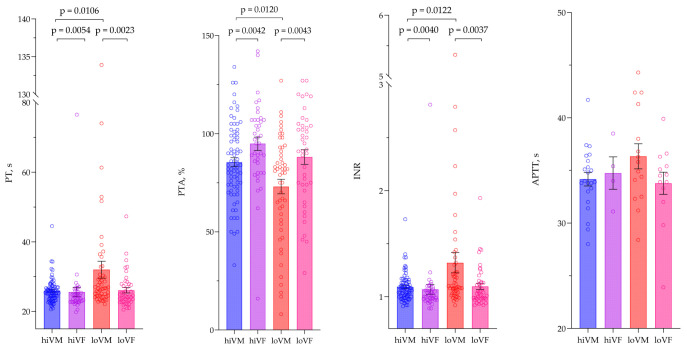
Comparison of coagulation index values between study groups. PTA—prothrombin time activity; PT—prothrombin time; INR—international normalized ratio; APTT—activated partial thromboplastin time. PT, PTA, INR—hiVM n = 75, hiVF n = 40, loVM n = 52, loVF n = 42. APTT—hiVM n = 22, hiVF n = 4, loVM n = 15, loVF n = 13. Lines connecting comparison groups with a *p*-value denoting the significant difference and pair-wise comparisons. Statistical analysis between investigated groups—two-sided Mann–Whitney U test (independent data); Wilcoxon signed-rank test used for evaluation of differences between first and second visits.

Significantly higher PT and INR, but lower PTA, were found in loVM compared with hiVM, *p* < 0.05. It was also found that hiVM had higher PT and INR rates and lower PTA than hiVF, *p* < 0.05. Significantly higher PT and INR, but lower PTA, were found in loVM compared with loVF, *p* < 0.05. However, due to the small sample size, APTT results did not differ between the groups. Data are presented in Figure 6.

When assessing whether coagulation tests met the normal limits applied in the laboratory, it was determined that only the APTT test had no statistically significant differences between the groups; however, the elevated values were found in 33% of loVM and in 25% of hiVF, while lower values were found in only 8% of the loVF group. In the PT test, only the elevated values were found in 60% of loVM, 46% of hiVM, 36% of loVF, and 13% of hiVF, *p* = 0.003. A similar tendency was found in PTA; the lower values were found in 38% of loVM, 21% of loVF, and 18% of hiVM, *p* < 0.001. INR was found to be elevated in 33% of loVM, 21% of loVF, and 14% of hiVM; while in the hiVF group, 5% had lower and 5% had higher INR results, *p* = 0.002. Even though the sample size in APTT tests was small, 33% of loVM, 25% of hiVF, and 4% of hiVM had higher results, while loVF had the same number of patients with both lower and higher APTT results.

### 3.5. Evaluation of Non-Invasive Hematological and Inflammation-Based Indices

FIB-4 index and APRI were significantly higher, while ALT/AST ratio, SII, AISI, and PNI were lower in loVM compared to hiVM, *p* < 0.05. AISI and SII results were significantly higher in the loVF group compared to hiVF, *p* < 0.05. FIB-4 and NLR results were significantly higher, while the ALT/AST ratio, SII, and PLR were lower in the loVM compared to loVF results, *p* < 0.05. The ALT/AST ratio and APRI results were significantly higher while AISI and NLR results significantly lower in hiVF compared to hiVM, *p* < 0.05.

**Figure 7:**
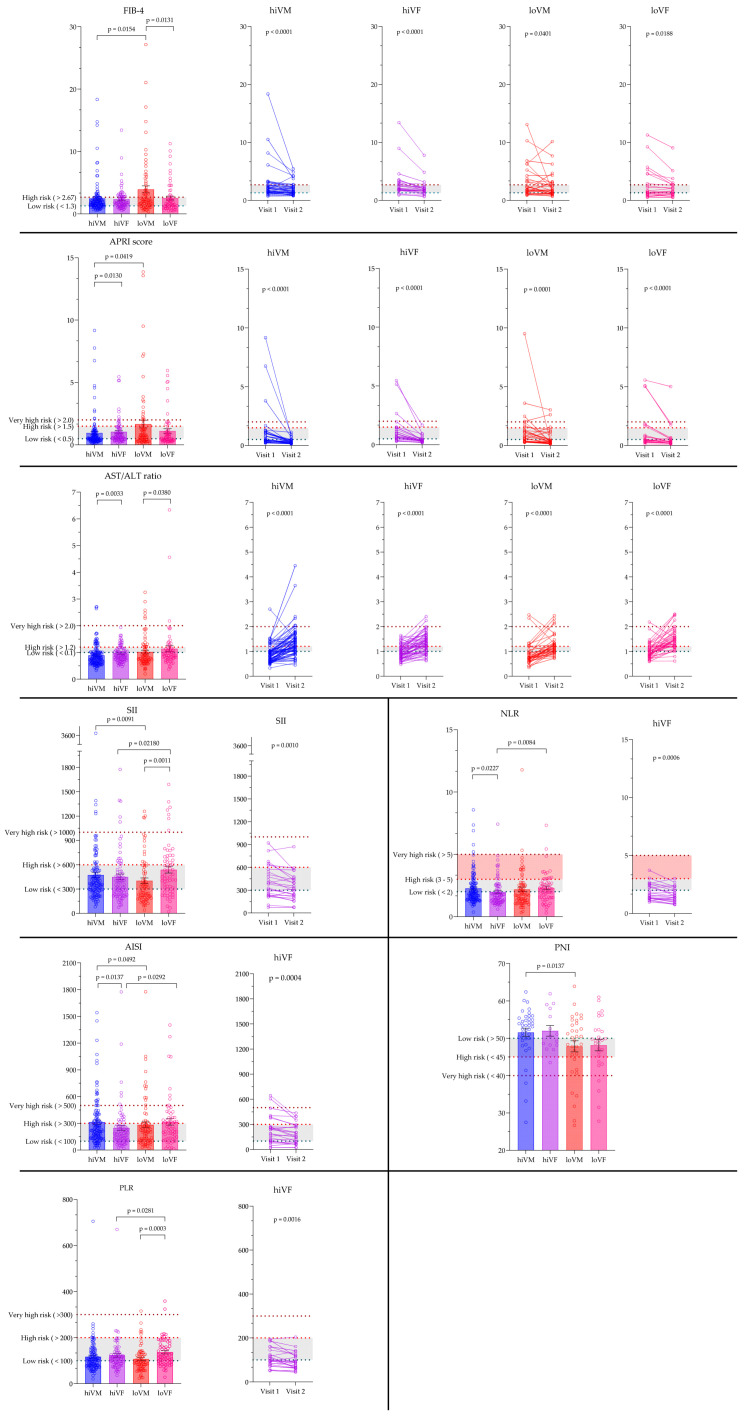
Comparison of non-invasive hematological and inflammation-based indices between the groups and visits. FIB-4, APRI: hiVM n = 119; hiVF n = 75; loVM n = 73; loVF n = 61. FIB-4, APRI Visit 1 vs. Visit 2: hiVM n = 36, hiVF n = 24, loVM n = 31, loVF n = 23. PLR, NLR, SII, AISI hiVM n = 123; hiVF n = 78, loVM n = 73, loVF n = 62. PLR, NLR, SII, AISI Visit 1 vs. Visit 2: hiVF n = 24. AST/ALT ratio hiVM n = 143, hiVF n = 80, loVM n = 73, loVF n = 61. AST/ALT ratio Visit 1 vs. Visit 2 hiVM n = 64, hiVF n = 51, loVM n = 38, loVF n = 40. PNI, HALP, NAR, PAR hiVM n = 39, hiVF n = 15, loVM n = 34, loVF n = 28. Lines connecting comparison groups with a *p*-value denoting the significant difference and pair-wise comparisons. Statistical analysis between investigated groups—two-sided Mann–Whitney U test (independent data); Wilcoxon signed-rank test used for evaluation of differences between first and second visits.

On the second visit, FIB-4 and APRI were significantly lower compared with the first visit value in all study groups, *p* < 0.05; meanwhile, the ALT/AST ratio was elevated on the second visit compared with the first visit in all study groups, *p* < 0.05. Only in the hiVF group the results of SII, AISI, PLR, and NLR on the second visit were lower compared to the first visit, *p* < 0.05. Data are presented in Figure 7.

## 4. Discussion

In this cohort of 367 HCV-infected patients stratified by gender and viremia, we observed clear, domain-specific patterns across hematology, biochemistry, coagulation, and derived indices. The loVM group paradoxically showed lower erythrocyte indices and platelet counts than the hiVM group, along with a worse coagulation profile (higher PT/INR, lower PTA) and increased levels of ALP, bilirubin, and AFP. Conversely, men consistently had higher GGT levels than women at similar viremia levels. Women demonstrated immune and enzyme differences: hiVF patients had higher AST and a higher AST/ALT ratio compared to other groups, as well as higher lymphocyte percentages and lower monocyte percentages. Inflammation-based ratios further differentiated the groups, with SII and PLR being highest in loVF, AISI highest in hiVM, and NLR lowest in loVF. Fibrosis-oriented measures mirrored these trends: FIB-4 was highest in loVM (compared to hiVM and loVF), and APRI was elevated in loVM and hiVF relative to hiVM. On follow-up after viral clearance, liver enzymes (AST, ALT, GGT) declined across all groups, fibrosis indices (FIB-4, APRI) decreased in every stratum, and selected inflammatory indices improved—most notably in hiVF—while WBC and lymphocyte counts increased in loVM. Collectively, these findings indicate that routine labs and their composite scores capture gender- and viremia-dependent disease burden and respond to treatment.

All of the analyzed non-invasive hematological and inflammation-based indices can be divided into three main groups: fibrosis-focused indices (FIB-4, APRI, AST/ALT ratio) that focus on structural liver damage; inflammation-based indices (NLR, PLR, SII, AISI, NAR) that focus on systemic immune/inflammatory balance; and nutritional/immune status indices (PNI, HALP, PAR) responsible for host nutritional and immunological reserve. Our study findings highlight the variability of HCV-related liver injury and systemic response, which seem to be heavily influenced by both gender and viral load [8,28]. The paradoxical connection between low viremia and worse hematological and fibrotic profiles in men suggests that viral replication is not the only factor causing liver damage; instead, host immune and inflammatory processes likely play a significant role. [29]. The higher AST/ALT ratio and lymphocyte dominance in women with high viremia indicate gender-specific immune regulation, aligning with reports of stronger antiviral immunity in females. The different patterns of inflammatory markers—SII and PLR peaking in low-viremia females, AISI in high-viremia males, and lowest NLR in high-viremia females—further support the idea that no single biomarker fully captures the spectrum of liver injury and immune activation [17]. Importantly, the consistent decreases in transaminases, fibrosis indices, and inflammatory ratios after treatment confirm the clinical relevance of these markers while also demonstrating their potential to monitor treatment response beyond just viral clearance [30].

Clinically, these results emphasize that routine laboratory parameters and their derived indices can provide valuable, non-invasive insights into the extent of liver injury and systemic involvement in chronic HCV. Our data confirm the utility of FIB-4 and APRI in differentiating advanced from minimal fibrosis, in line with prior reports that established both as reliable alternatives to biopsy [12,13]. Similarly, the observed patterns in NLR, PLR, and SII extend previous findings linking inflammation-based ratios with fibrosis progression and HCC risk [14,18,21]. The gender-related differences we identified complement earlier studies, which have shown stronger immune-mediated control of HCV in women, as reflected by higher lymphocyte ratios and a more favorable inflammatory profile despite ongoing viral replication [31,32]. By contrast, the paradoxical association of low viremia with worse hematologic and fibrotic measures in men is less frequently reported, underscoring the need for careful interpretation of viral load in clinical practice [33,34]. Overall, these results indicate that combining fibrosis indices, inflammatory ratios, and nutritional and immune markers in a panel-based approach could be more effective in assessing disease severity and monitoring patients than relying solely on viral load.

To improve clinical interpretability and translational relevance, we suggest a concise laboratory index panel that combines key biochemical and hematological markers reflecting different aspects of liver disease. Serum ALT and AST indicate hepatocellular injury, while platelet count is a sensitive marker of portal hypertension and fibrotic remodeling. Albumin, bilirubin, and INR together describe liver synthetic and excretory functions, offering a clear overview of remaining liver capacity. Derived indices like FIB-4 and APRI aggregate these routine tests into validated, non-invasive indicators of fibrosis severity. The PLR measures systemic inflammation, which can impact disease progression and post-treatment outcomes recovery.

In a practical clinical setting, abnormal results in this core panel can help prioritize patients for further evaluation, such as elevated FIB-4 or APRI scores leading to transient elastography or hepatology referral. Conversely, low albumin or prolonged INR would indicate the need to assess hepatic reserve and nutritional state. This efficient, evidence-based method strikes a balance between detailed analysis and clinical usefulness, enabling the integration of laboratory markers into real-world care pathways without overstating their diagnostic purpose.

The paradoxical finding that males with lower baseline viremia showed worse biochemical profiles likely reflects complex immune–fibrotic interactions rather than milder disease activity. Evidence shows that low viral replication can coexist with advanced or “burnt-out” fibrosis, where hepatocellular inflammation decreases while structural damage remains [35]. Post-DAA studies show persistent T-cell exhaustion and incomplete immune restoration despite sustained virologic response [36], alongside residual inflammatory and metabolic dysregulation [37]. Fibrosis regression after clearance remains variable, with some patients demonstrating ongoing collagen turnover [38]. Collectively, these data suggest that viral load alone underestimates hepatic and immunologic activity and that post-treatment laboratory indices incorporating inflammatory and fibrotic surrogates may better capture residual disease dynamics.

A key strength of this study is the simultaneous evaluation of various hematological, biochemical, and derived indices across gender- and viremia-defined subgroups, both before and after viral clearance. This comprehensive approach allowed us to show that markers of fibrosis, inflammation, and nutritional/immune status not only differentiate patient profiles at baseline but also change dynamically with treatment. Importantly, the longitudinal design reinforces the evidence that improvements in these indices correlate with virological cure, supporting their potential use as additional monitoring tools in clinical practice. Future research should confirm these findings in larger, multicenter cohorts and investigate whether adding such indices to risk models could enhance prognostication, guide follow-up strategies, and ultimately personalize care for patients with chronic HCV.

Growing evidence emphasizes the complex link between chronic HCV infection and autoimmune phenomena. Recent data show an increased prevalence of autoimmune serological markers in individuals with chronic HCV, supporting a possible immunopathological connection between HCV-related liver damage and autoimmune hepatitis [38]. Furthermore, several reports have described de novo or unmasked autoimmune liver disorders following rapid viral clearance induced by direct-acting antivirals, underscoring the need for continued immunological surveillance even after virologic cure. The dynamic interplay between viral persistence, immune reconstitution after DAA therapy, and host autoimmunity represents an emerging field of clinical relevance. Within this context, immune–inflammatory and nutritional indices such as PLR, NLR, SII, and HALP may offer additional insight into subclinical immune dysregulation, complementing conventional hepatic biomarkers and informing hypotheses about post-treatment immune adaptation.

This study’s limitations may include small sample sizes in some subgroups, especially during the second visit, which restricts the generalizability of the findings. Additionally, this study did not consider other potentially important factors, such as alcohol use, body mass index, or comorbidities, since the laboratory results were based on known gender, age, and viremia level. Future research should include these factors and deepen the understanding of how gender interacts with the immune system in HCV infection. It should also expand the analysis of immunological markers and incorporate additional clinical variables (e.g., alcohol consumption habits, body mass index, comorbidities) to better examine differences between groups.

## 5. Conclusions

HCV infection affects hematological, biochemical, and coagulation parameters in a gender- and viremia-dependent manner. Non-invasive indices such as FIB-4, APRI, SII, and PLR provide valuable complementary information on fibrosis and systemic inflammation. Incorporating these indices into clinical practice may enhance patient evaluation and support the development of tailored monitoring strategies that extend beyond standard laboratory testing.

## Figures and Tables

**Table 1:** Non-invasive hematological and inflammation-based indices, their formulas for calculations, and possible clinical relevance in HCV patients.

Index	Cut-Off Values (Risk Zones)	Formula	Clinical Relevance in HCV Patients	
**FIB-4** (Fibrosis-4 Index)	<1.45 → low risk; 1.45–3.25 → intermediate; >3.25 → high risk of advanced fibrosis	(Age [years] × AST [U/L])/(PLT [10^9^/L × √ALT[U/L])	Non-invasive assessment of liver fibrosis reduces the need for biopsy	[5,12,17]
**PLR** (Platelet-to-Lymphocyte Ratio)	<100 → low; 100–200 → intermediate; >200 → high inflammation/fibrosis risk	PLT [×10^9^/L]/LYMPH [×10^9^/L]	Reflects systemic inflammation, associated with fibrosis progression in HCV	[5,18,19]
**AST/ALT ratio**	<1 → usually non-advanced disease; >1 → advanced fibrosis or cirrhosis	AST [U/L]/ALT [U/L]	Simple marker for disease severity; ratio >1 often indicates cirrhosis	[20]
**NLR** (Neutrophil-to-Lymphocyte Ratio)	<2 → normal; 2–4 → intermediate; >4 → high inflammatory risk	NEUTRO [×10^9^/L]/LYMPH [×10^9^/L]	An indicator of systemic inflammation, it predicts the progression of fibrosis and the risk of HCC	[21,22]
**APRI** (AST to Platelet Ratio Index)	<0.5 → low risk; 0.5–1.5 → intermediate; >1.5 → high risk of significant fibrosis	[(AST [IU/L]/ upper limit of normal AST [IU/L])/PLT [×10^9^/L]] × 100	Widely used non-invasive tool for fibrosis and cirrhosis	[13,19]
**SII** (Systemic Immune-Inflammation Index)	<500 → low; 500–1000 → intermediate; >1000 → high risk	(PLT [×10^9^/L] × NEUTRO [×10^9^/L])/LYMPH [×10^9^/L]	Captures systemic inflammation and immune status, linked to fibrosis progression	[14,19]
**AISI** (Aggregate Index of Systemic Inflammation)	No universal cut-offs; higher values = worse prognosis	(NEUTRO [×10^9^/L ] × MONO [×10^9^/L ] × PLT [×10^9^/L])/LYMPH [×10^9^/L]	Comprehensive inflammation index, explored for HCV outcomes	[19]
**PNI** (Prognostic Nutritional Index)	>45 → good; 40–45 → moderate; <40 → poor nutritional/immune status	10 × ALB [g/L] + 0.005 × LYMPH [×10^9^/L]	Reflects nutritional and immune reserve; predictive of chronic liver disease	[23,24]
**HALP** (Hemoglobin, Albumin, Lymphocyte, Platelet Index)	>30 → favorable; <30 → unfavorable prognosis	(HGB [g/L] × ALB [g/L] × LYMPH [×10^9^/L])/PLT [×10^9^/L]	Integrates inflammation and nutrition, associated with outcomes in HCV	[22]
**PAR** (Platelet-to-Albumin Ratio)	Higher ratio = worse fibrosis; no fixed cut-offs yet	PLT [×10^9^/L]/ALB [g/L]	Reflects impaired albumin synthesis + thrombocytopenia in liver damage	[25,26]
**NAR** (Neutrophil-to-Albumin Ratio)	>0.15–0.20 → higher risk (study-dependent)	NEUTRO [×10^9^/L]/ALB [g/L]	Combines inflammation and liver synthetic function, linked with poor outcomes	[27]

ALB—albumin; AST—aspartate aminotransferase; ALT—alanine transaminase; HGB—hemoglobin; LYMPH—lymphocytes; NEUTRO—neutrophils; PLT—platelets; MONO—monocytes.

**Table 2:** Distribution of high and low viremia by gender and age, n = 367.

	High Viremia		Low Viremia	
n	231		136	
Viremia, IU/ml	≥800,000		<800,000	
Gender (males/females)	149	82	74	62
Age (mean ± SD)	53.48 ± 10.29	58.32 ± 10.63	55.01 ± 10.57	55.40 ± 13.78
Viremia, Mean (95% CI of mean)	8,867,906 (7,574,998–10,160,814)	8,780,378 (5,076,277–12,484,479)	204,336 (163,653–245,019)	255,093 (206,549–303,637)
Time between I and II visit, mean ± SD (HCV RNA = 0 IU/mL)	17 ± 4			

**Table 3:** Distribution of study groups by genotype, n = 367.

	1	1a	1a/1b	1b	2	2a/2c	3	3a	Total
	n (%)	n (%)	n (%)	n (%)	n (%)	n (%)	n (%)	n (%)	
**hiVM**	0	36	1	49	3	18	0	42	149
	(0.0)	(24.16)	(0.67)	(32.89)	(2.01)	(12.08)	(0.00)	(28.19)	
**hiVF**	0	7	0	45	2	6	1	21	82
	(0.00)	(8.54)	(0.00)	(54.88)	(2.44)	(7.32)	(1.22)	(25.61)	
**loVM**	3	10	2	31	1	5	1	21	74
	(4.05)	(13.51)	(2.70)	(41.89)	(1.35)	(6.76)	(1.35)	(28.38)	
**loVF**	0	2	0	30	6	2	0	22	62
	(0.00)	(3.23)	(0.00)	(48.99)	(9.68)	(3.23)	(0.00)	(35.48)	
**Total**	**3**	**55**	**3**	**155**	**12**	**31**	**2**	**106**	**367**
	**(0.82)**	**(14.99)**	**(082)**	**(42.23)**	**(3.27)**	**(8.45)**	**(0.54)**	**(28.88)**	

hiVM—high viremia males; hiVF—high viremia females; loVM—low viremia males; loVF—low viremia females.

## Data Availability

This article includes all the data presented in this study.

## Abbreviations

The following abbreviations are used in this manuscript:

AFP	α-fetoprotein
AISI	Aggregate index of systemic inflammation
ALB	Albumin
ALP	Alkaline phosphatase
ALT	Alanine aminotransferase
APRI	Aspartate aminotransferase to platelet ratio index
APTT	Activated Partial Thromboplastin Time
AST	Aspartate Aminotransferase
CREA	Creatinine
EASL	European Association for the Study of the Liver
FIB-4	Fibrosis-4 index
GGT	γ-glutamyl transferase
HALP	Hemoglobin, albumin, lymphocytes, platelet index
HCC	Hepatocellular carcinoma
HCT	Hematocrit
HCV	Hepatitis C virus
HGB	Hemoglobin
hiVF	High viremia females
hiVM	High viremia males
INR	International Normalized Ratio
loVF	Low viremia females
loVM	Low viremia males
LUHS	Lithuanian University of Health Sciences
LYMPH	Lymphocytes
MONO	Monocytes
MPV	Mean platelet volume
NAR	Neutrophil–albumin ratio
NLR	Neutrophil–lymphocyte ratio
PAR	Platelet–albumin ratio
PCT	Plateletcrit
PLR	Platelets–lymphocytes ratio
PLT	Platelets
PNI	Prognostic nutritional index
PTA	Prothrombin time activity
PT	Prothrombin time
RBC	Red blood cells
SD	Standard deviation
SEM	Standard error of mean
SII	Systemic immune-inflammation index
WBC	White blood cells
WHO	World Health Organization

## Appendix A

**Table A1:** Normal ranges of hematological, biochemical, and coagulation tests of the Clinic of Laboratory Medicine at Hospital of LUHS Kauno Klinikos.

	Males	Females
Hematological tests		
RBC, ×10^12^/L	4.32–5.72	3.9–5.03
HGB, g/L	135–175	120–155
HCT, %	38.8–50	34.9–44.5
WBC, ×10^9^/L	3.6–10.2	3.8–11.8
Lymphocytes, ×10^9^/L	1–3.2	1.1–3.1
Lymphocytes, %	15.2–43.3	16–45.9
Monocytes, ×10^9^/L	0.3–1.1	0.2–0.9
Monocytes, %	5.5–13.7	4.3–10.9
PLT, ×10^9^/L	152–348	179–408
MPV, fL	7.4–11.4	7.9–10.8
PCT, %	0.15–0.35	0.15–0.35
Biochemical tests		
AST, IU/L	0–50	0–35
ALT, IU/L	1–50	0–35
CREA, μmol/L	59–104	45–84
ALP, IU/L	30–120	
AFP, kU/L		
GGT, IU/L	1–39	
DBIL, μmol/L	0–3.4	
TBIL, μmol/L	5–21	
ALB, g/L	35–52	
Coagulation tests		
PT, s	19.8–25.8	
PT, %	70–130	
INR	0.9–1.2	
APTT, s	28–38	

**Table A2:** Full statistical description of each group. Groups were compared using the non-parametric Kruskal–Wallis test, with significant differences at *p* < 0.05. * indicates results with different normal ranges based on age and gender; these results pair-wise were evaluated just in different viremia levels, but not gender. Data presented: mean, standard deviation, standard error of mean, 95% CI lower and upper values of the medium.

Statistical Parameters		hiVM		hiVF		loVM		loVF		*p*
RBC, ×10^12^/L										
Mean	n	4.72	121	4.52	78	4.55	73	4.43	62	**0.0001 ***
SD	CI Lower	0.507	4.62	0.332	4.38	0.574	4.51	0.484	4.38	
SEM	CI Upper	0.0461	4.90	0.0376	4.60	0.0672	4.78	0.0615	4.61	
HGB, g/L										
Mean	n	149	121	139	78	144	73	134	62	**<0.0001 ***
SD	CI Lower	15.1	146	10.7	138	18.1	142	16.1	133	
SEM	CI Upper	1.37	156	1.21	143	2.12	153	2.04	140	
HCT, %										
Mean	n	43.3	121	40.0	78	41.8	73	39.4	62	**<0.0001 ***
SD	CI Lower	4.11	42.8	5.07	40.0	4.91	41.2	4.40	38.9	
SEM	CI Upper	0.373	45.0	0.574	41.4	0.575	43.9	0.558	41.3	
WBC, ×10^9^/L										
Mean	n	5.964	121	6.247	78	6.409	73	6.251	62	0.0841 *
SD	CI Lower	2.014	5.020	2.140	5.440	2.245	5.570	2.050	5.420	
SEM	CI Upper	0.1446	5.630	0.2423	6.340	0.2627	6.730	0.2603	6.210	
Lymphocytes, %										
Mean	n	29.93	121	32.77	78	31.45	73	29.97	62	0.0861 *
SD	CI Lower	9.293	33.1	8.844	30.1	10.59	27.5	9.667	25.8	
SEM	CI Upper	0.8448	27.8	1.001	36.8	1.239	34.5	1.228	31.6	
Monocytes, %										
Mean	n	9.650	121	8.731	78	10.46	73	9.053	62	**0.0058 ***
SD	CI Lower	2.976	8.70	2.134	8.00	3.290	9.10	2.967	7.70	
SEM	CI Upper	0.2706	10.0	0.2416	8.90	0.3851	11.3	0.3768	9.30	
Lymphocytes, ×10^9^/L										
Mean	n	2.04	121	2.00	78	1.97	73	1.80	62	0.3238 *
SD	CI Lower	1.03	1.80	0.764	1.80	0.874	1.70	0.646	1.50	
SEM	CI Upper	0.0936	2.10	0.0865	2.00	0.102	2.10	0.0820	2.00	
Monocytes, ×10^9^/L										
Mean	n	0.631	121	0.527	78	0.649	73	0.544	62	**0.0006 ***
SD	CI Lower	0.224	0.600	0.196	0.400	0.256	0.600	0.188	0.500	
SEM	CI Upper	0.0204	0.700	0.0222	0.600	0.0300	0.600	0.0239	0.600	
PLT, ×10^9^/L										
Mean	n	206	121	219	78	189	73	228	62	**0.0013 ***
SD	CI Lower	66.1	191	66.0	202	76.5	165	79.4	212	
SEM	CI Upper	6.01	213	7.48	228	8.96	203	10.1	257	
MPV, fL										
Mean	n	8.82	121	8.75	78	8.74	73	8.71	62	0.8955 *
SD	CI Lower	1.09	8.50	0.818	8.30	1.07	8.20	0.911	8.30	
SEM	CI Upper	0.0990	9.00	0.0927	9.00	0.125	8.90	0.116	9.00	
PCT, %										
Mean	n	0.178	121	0.190	78	0.163	73	0.194	62	**0.0007 ***
SD	CI Lower	0.0481	0.170	0.0576	0.170	0.0637	0.150	0.0597	0.190	
SEM	CI Upper	0.00437	0.180	0.00652	0.200	0.00746	0.170	0.00758	0.220	
AST, IU/L										
Mean	n	75.9	143	67.9	80	87.2	73	65.0	61	0.0509 *
SD	CI Lower	63.0	50.0	44.0	44.0	77.9	51.0	58.2	36.0	
SEM	CI Upper	5.27	63.0	4.92	65.0	9.12	74.0	7.45	54.0	
ALT, IU/L										
Mean	n	98.0	144	78.3	80	96.6	73	66.2	61	**0.0139 ***
SD	CI Lower	79.2	57.8	55.8	50.6	74.1	60.4	49.4	43.1	
SEM	CI Upper	6.60	88.2	6.24	75.2	8.68	100	6.33	58.8	
ALP, IU/L										
Mean	n	73.1	111	73.4	59	90.6	59	76.6	50	0.2075
SD	CI Lower	27.0	63.0	22.2	63.0	45.2	67.0	26.4	65.0	
SEM	CI Upper	2.56	74.0	2.90	76.0	5.89	89.0	3.73	82.0	
GGT, IU/L										
Mean	n	98.50	128	52.48	67	103.5	61	70.12	50	**0.0002**
SD	CI Lower	133.9	40.00	53.24	24.00	118.1	39.00	89.76	21.00	
SEM	CI Upper	11.83	62.00	6.505	46.00	15.12	99.00	12.69	49.00	
CREA, μmol/L										
Mean	n	77.22	88	62.72	54	74.93	55	63.07	41	**<0.0001 ***
SD	CI Lower	13.09	73.00	11.88	58.00	17.76	68.00	20.84	55.00	
SEM	CI Upper	1.396	78.00	1.617	63.00	2.395	79.00	3.254	64.00	
Total bilirubin, μmol/L										
Mean	n	15.19	73	12.59	36	28.14	45	13.35	45	**0.0235**
SD	CI Lower	9.723	11.14	5.014	10.17	34.32	12.09	9.273	9.030	
SEM	CI Upper	1.138	14.58	0.8357	14.46	5.116	16.69	1.414	13.44	
Direct bilirubin, μmol/L										
Mean	n	3.214	49	2.496	20	12.69	34	5.602	31	0.0744
SD	CI Lower	2.105	2.350	1.436	1.740	20.93	2.440	15.62	1.770	
SEM	CI Upper	0.3008	3.110	0.3210	3.140	3.590	4.500	2.805	3.470	
AFP, kU/L										
Mean	n	6.603	29	5.923	18	26.77	25	29.50	13	**0.0315**
SD	CI Lower	11.05	2.850	3.502	3.600	64.79	4.830	93.34	2.320	
SEM	CI Upper	2.052	4.540	0.8255	7.830	12.96	6.170	25.89	5.300	
ALB, g/L										
Mean	n	41.93	42	42.26	16	38.88	35	39.42	28	**0.0273**
SD	CI Lower	4.869	41.50	3.089	39.70	6.262	39.90	5.532	38.10	
SEM	CI Upper	0.7514	44.00	0.7723	44.30	1.058	42.00	1.045	42.70	
PT, s										
Mean	n	25.93	75	25.57	40	32.00	52	26.08	42	**0.0002**
SD	CI Lower	3.533	24.60	8.521	23.00	17.57	25.10	5.055	23.50	
SEM	CI Upper	0.4080	26.30	1.347	24.80	2.436	28.60	0.7800	25.80	
PTA, %										
Mean	n	85.51	75	94.88	40	73.06	52	88.10	42	**0.0004**
SD	CI Lower	19.67	80.00	21.46	89.00	26.66	66.00	24.32	77.00	
SEM	CI Upper	2.271	90.00	3.393	104.0	3.697	84.00	3.752	102.0	
INR										
Mean	n	1.09	75	1.07	40	1.32	52	1.10	42	**0.0004**
SD	CI Lower	0.131	1.04	0.291	0.990	0.688	1.07	0.193	0.990	
SEM	CI Upper	0.0151	1.10	0.0461	1.05	0.0954	1.19	0.0297	1.11	
APTT, s										
Mean	n	34.2	22	34.8	4	36.3	15	33.8	13	0.4536
SD	CI Lower	2.99	33.0	3.08	31.1	4.60	32.5	3.81	32.0	
SEM	CI Upper	0.637	35.9	1.54	38.5	1.19	41.3	1.06	36.3	
FIB-4										
Mean	n	2.49	119	2.37	75	3.95	73	2.54	61	0.0604
SD	CI Lower	2.66	1.54	1.88	1.60	4.77	1.74	2.43	1.33	
SEM	CI Upper	0.244	2.01	0.217	2.34	0.558	2.88	0.311	2.15	
APRI										
Mean	n	0.960	119	1.05	75	1.67	73	1.12	61	0.1182
SD	CI Lower	1.36	0.470	1.03	0.590	2.67	0.560	1.39	0.420	
SEM	CI Upper	0.124	0.700	0.119	0.930	0.312	1.08	0.178	0.840	
PLR										
Mean	n	117	123	124	78	107	73	137	62	**0.0027**
SD	CI Lower	70.9	94.4	75.8	98.5	56.0	85.0	59.2	107	
SEM	CI Upper	6.40	111	8.59	118	6.51	110	7.51	153	
NLR										
Mean	n	2.27	123	2.00	78	2.18	73	2.32	62	0.0687
SD	CI Lower	1.28	1.71	1.11	1.39	1.56	1.46	1.16	1.95	
SEM	CI Upper	0.116	2.04	0.126	1.88	0.182	2.18	0.148	2.55	
AST/ALT ratio										
Mean	n	0.894	143	0.959	80	1.02	73	1.14	61	**0.0075**
SD	CI Lower	0.391	0.750	0.286	0.850	0.595	0.730	0.884	0.840	
SEM	CI Upper	0.0324	0.880	0.0299	1.01	0.0678	0.920	0.112	1.05	
SII										
Mean	n	472	123	450	78	402	73	540	61	**0.0082**
SD	CI Lower	383	344	314	291	294	229	337	399	
SEM	CI Upper	34.6	426	35.6	434	34.1	365	42.8	591	
AISI										
Mean	n	313	123	248	78	283	73	316	61	0.0769
SD	CI Lower	269	207	252	150	290	137	280	189	
SEM	CI Upper	24.2	272	28.6	233	33.7	254	35.6	321	
PNI										
Mean	n	51.6	39	52.0	15	47.8	34	48.2	28	0.0548
SD	CI Lower	6.85	50.6	5.50	48.2	8.70	45.8	7.88	46.2	
SEM	CI Upper	1.10	54.8	1.42	58.0	1.49	52.5	1.49	51.8	
HALP										
Mean	n	66.7	39	61.0	15	61.2	34	53.9	28	0.2528
SD	CI Lower	32.8	46.3	27.2	46.1	25.4	46.8	33.0	39.4	
SEM	CI Upper	5.26	69.2	7.02	67.8	4.36	79.5	6.23	59.2	
NAR										
Mean	n	0.100	39	0.0847	15	0.108	34	0.0875	28	0.4963
SD	CI Lower	0.0437	0.0700	0.0309	0.0600	0.105	0.0600	0.0551	0.0700	
SEM	CI Upper	0.00700	0.110	0.00798	0.0900	0.0179	0.110	0.0104	0.0900	
PAR										
Mean	n	5.05	39	4.69	15	4.56	34	4.88	28	0.4486
SD	CI Lower	1.86	4.44	1.17	3.88	1.99	3.71	1.87	3.93	
SEM	CI Upper	0.297	5.27	0.303	5.15	0.341	4.98	0.353	5.88	
